# Assessing perinatal depression as an indicator of risk for pregnancy-associated cardiovascular disease

**DOI:** 10.5830/CVJA-2015-087

**Published:** 2016

**Authors:** Lauren Nicholson, Sandrine Lecour, Karen Sliwa, Sonja Wedegärtner, Ingrid Kindermann, Michael Böhm

**Affiliations:** Hatter Institute for Cardiovascular Research in Africa and MRC Inter-University Cape Heart group, Department of Medicine, University of Cape Town, South Africa; Hatter Institute for Cardiovascular Research in Africa and MRC Inter-University Cape Heart group, Department of Medicine, University of Cape Town, South Africa; Hatter Institute for Cardiovascular Research in Africa, and IDM, Department of Medicine, Faculty of Health Sciences, University of Cape Town, South Africa; Soweto Cardiovascular Research Unit, University of the Witwatersrand, Johannesburg; Inter-Cape Heart Group, Medical Research Council South Africa, Cape Town, South Africa; Clinic for Internal Medicine III, Cardiology, Angiology and Intensive Care Medicine, University Hospital of Saarland, Homburg/Saar, Germany; Clinic for Internal Medicine III, Cardiology, Angiology and Intensive Care Medicine, University Hospital of Saarland, Homburg/Saar, Germany; Clinic for Internal Medicine III, Cardiology, Angiology and Intensive Care Medicine, University Hospital of Saarland, Homburg/Saar, Germany

**Keywords:** cardiovascular disease in pregnancy, peripartum cardiomyopathy, depression in pregnancy

## Abstract

Cardiovascular conditions associated with pregnancy are serious complications. In general, depression is a well-known risk indicator for cardiovascular disease (CVD). Mental distress and depression are associated with physiological responses such as inflammation and oxidative stress. Both inflammation and oxidative stress have been implicated in the pathophysiology of CVDs associated with pregnancy. This article discusses whether depression could represent a risk indicator for CVDs in pregnancy, in particular in pre-eclampsia and peripartum cardiomyopathy (PPCM).

## Abstract

The physiological changes associated with pregnancy, such as increased oxidative stress and circulatory changes, place a burden on the cardiovascular system of pregnant women (Fig. 1).^1^ Cardiovascular conditions associated with pregnancy, such as peripartum cardiomyopathy (PPCM) and pre-eclampsia, could result in serious cardiovascular complications.^2^,^3^

**Fig. 1.**
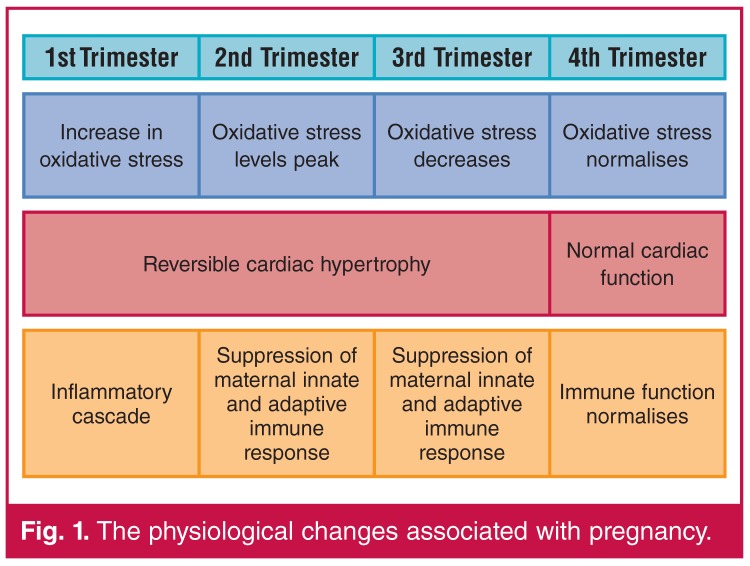
The physiological changes associated with pregnancy.

Psychosocial factors, for example depression, are increasingly being recognised as risk indicators for cardiovascular diseases such as ischaemic heart disease.^4^ Mental disorders such as anxiety and depression are the third leading burden of disease in women globally.^5^ Women of childbearing age have the highest prevalence of psychiatric disorders, in particular, anxiety and mood disturbances.^6^,^7^

Previous studies have explored the association between depression and cardiovascular disease (CVD),^4^,^8^ and have demonstrated that depression is a risk factor for CVD and increases both morbidity and mortality rates.^8^ This article discusses the potential contribution of depression during the peripartum period to the pathophysiology of CVD in pregnancy.

## Physiological adaptations in pregnancy

Major compensatory changes are made by the maternal heart to accommodate the demands of pregnancy and lactation.^9^ In pregnancy, the foreign material of the foetus is not rejected by the maternal immune system,^10^ as increased oxidative stress during the first trimester prevents this rejection.^11^ During pregnancy women experience a reversible adaptive cardiac hypertrophy (Fig. 1) and reduced relaxation of diastolic function, whereas in healthy women this regresses to normal following childbirth.^12^

## Increase in oxidative stress during pregnancy

In the first trimester, oxidative stress, which is an increased production of reactive oxygen species compared to antioxidant defence mechanisms,^13^ regulates the invasion of foreign trophoblastic material in the maternal body.^11^ These oxidative stress mechanisms also control normal and pathological embryogenesis.^14^ The hormone oestrogen mediates regulation of the balance between pro-oxidative and anti-oxidative molecules guarding this process.^14^

An increase in oxidative stress during pregnancy can be characterised by enhanced lipid peroxidation and the circulation of lipid hydroperoxides.^14^ The increase in oxidative stress in healthy women peaks around the second trimester.^15^ When the pregnancy becomes advanced, disruption in this oxidative balance can lead to inappropriate activation of the inflammatory cascade, which produces harmful effects, including premature labour and complications such as pre-eclampsia.^14^

## The inflammatory response in pregnancy

At the start of pregnancy an inflammatory cascade is activated, which allows the formation and invasion of the foreign trophoblastic material into the maternal tissues.^16^ Medawar *et al.* proposed a model by which suppression of the mother’s immune system allows invasion of the foreign material.^10^ This model suggests that maternal lymphocyte suppression allows this invasion.^10^

The immune system uses two basic components: the non-specific inbuilt innate immune response and the specific ‘learned’ adaptive immune system.^17^ The innate immune system is primitive. Its primary function is to differentiate self from non-self and it only copes with the most fundamental immune challenges, such as pathogens.^17^ The innate immune system presents antigens in association with major histocompatibility complex (MHC) class I and II molecules to the lymphocytes. The more specific adaptive immune system has a delayed response. The cells learn and develop an acquired defence against external threats.^17^

Human pregnancy presents a unique challenge to the immune system.^18^ The uterus is surrounded by a mucosal barrier, the decidua.^18^ It is, however, not impenetrable to the maternal immune system.^19^ The trophoblast cells do not express MHC class I or II molecules, thereby escaping the maternal innate immune response. Imbalances in the innate immune response in the placenta and decidua have been implicated in the development of pre-eclampsia.^20^

Adaptive immune responses are suppressed by placental products such as prostaglandins and interleukins 4, 6 and 10 (IL-4, IL-6 and IL-10). IL-6 is an important cytokine in the immune inflammatory response in adaptive immunity during pregnancy.^21^ Excessive IL-6 response has been implicated in pathological conditions of pregnancy, such as miscarriage and pre-eclampsia.^21^

## Women and cardiovascular disease

Cardiovascular disease, once thought to be a ‘male problem’, is now recognised as equally affecting women.^22^ The American Heart Association published the first women-specific clinical recommendations in 1999, which led to an increase in awareness and prevention of CVD in women.^22^ The rate of deaths resulting from CVD are however still increasing, due to diseases of lifestyle leading to an increase in hypertension and diabetes.^22^ Around 81% of CVD deaths in women occur in lower-income countries.^22^

In women with pre-existing heart disease, changes in the circulatory system during pregnancy can cause decompensation or death of the foetus or mother.^23^ Atkins and colleagues investigated the differences in risk factors in American women of Caucasian and African racial groups.^24^ Caucasian women have been found to have higher rates of hyperhomocysteinaemia and higher body mass index (BMI). African women were found to have an increase in blood pressure, BMI and iron-deficiency anaemia. Physiological changes during pregnancy in women with no known pre-existing CVD may lead to the development of PPCM and pre-eclampsia.

Pre-eclampsia occurring in late or early pregnancy is characterised by hypertension, oedema and the presence of protein in the urine.^3^ Hypertensive disorders are the most frequent complication in pregnancy and cause of maternal death in Africa.^12^ There have also been limited insights into the exact pathophysiological mechanisms of the disease.^25^ A suggested pathophysiological mechanism is an increase in oxidative stress during pregnancy.^26^

PPCM presents in the final month of pregnancy and during the first five months postpartum.^2^ Distinguished from other forms of cardiomyopathy by its rapid development in the peripartum period, the exact mechanism of PPCM is not well understood.^27^ In countries with large populations of African descent, such as South Africa and Haiti, the prevalence is higher, with one in 1 000 and one in 299 births, respectively.^28^ More epidemiological studies are needed to fully determine the prevalence rates in Europe and Asia.^1^

Studies have suggested that an increase in oxidative stress during pregnancy leads to the cleavage of the breastfeeding hormone, prolactin, into a 16-Kda pro-apoptotic, which may contribute to the development of PPCM.^28^ Increases in pro-inflammatory cytokines such as C-reactive protein (CRP) have also been suggested to contribute to the condition.^29^

## Depression as a risk factor for cardiovascular disease

Since the time of the ancient Greeks, affective dispositions have been thought to be associated with physical disease.^30^ The World Health Organisation (WHO) estimates that, by the year 2030, mental disorders will rise to first place in hospitalisation morbidity, overtaking road traffic accidents and heart disease.^31^

Depression is known to be a risk factor for the development of CVD, as well as a predictor of poor prognosis following a cardiac event.^8^ Established risk factors, such as hypercholesterolaemia, hypertension and smoking, leave unexplained inconsistencies in ischaemic heart disease data.^32^ It has been suggested that psychosocial factors may account for these differences.^32^ The mental and physiological changes of a depressive individual may also negatively affect the course of CVD.^8^ The decrease in the depressive patient’s motivation and inability to function in day-to-day tasks, as well as fear of side effects, may result in non-compliance with medical recommendations.^8^ Depression also increases the incidence of other risk indicators, such as smoking and hypertension.^8^

Previous animal and human models have suggested links in the pathways between depression and physiological responses, such as nervous system activation, an increase in inflammation, changes in sleep patterns and cardiac rhythm disturbances.^8^,^30^ Rosengren and colleagues investigated the association of psychosocial factors with the risk of myocardial infarction.^4^ This study found that patients with myocardial infarction reported high psychosocial stress factors, such as depression and financial and work stress, compared to healthy individuals.^4^ The increased risk for CVD is potentially due to the physiological response to these psychosocial stressors.

Depression has been shown to increase inflammatory cytokines, which are known to contribute to CVD.^33^ Inflammatory markers such as CRP, tumour necrosis factor-α (TNF-α) and IL-6 have been associated with an increased risk for CVD.^33^ Vaccarino and colleagues investigated depression, inflammation and cardiovascular outcomes of women.^33^ Women with established depression had 70% higher CRP levels than women without depression.^33^ The study also suggested that the association between depression and CVD cannot be explained by inflammation alone.^33^ Kamarck and colleagues performed a prospective study to determine the directionality of the association between depression and inflammatory markers in both men and women.^1^ The study found that only BMI had a greater association with increased CRP and IL-6 than depression.^1^

## Depression during pregnancy and postnatal depression

Perinatal depression is a serious and prevalent mental health condition occurring towards the end of pregnancy and up until the first year postpartum.^34^,^35^ Depression is disabling for women and is most common during the childbearing years.^35^ Postpartum depression refers to the depressive disorders occurring during the postpartum period, up until the first year following childbirth.^35^

In developed countries, studies have shown that the prevalence of postpartum depression is around 10–15%.^36^ However, in developing regions, the proportion is often double that of developed regions. A study in western Nigeria reported the incidence of perinatal depression during pregnancy to be 31.3%.^37^

A South African study found that 32% of the perinatal women screened for maternal depression qualified for referral to counselling.^5^ The case study found that there is a deficiency in screening for depression in primary healthcare in South Africa and many cases are not identified.^5^ They used the Edinburgh Postnatal Depression Scale (EDPS) as a screening tool.^5^ This is a validated 10-item questionnaire used for screening for a probable diagnosis of depression, both pre- and postpartum.^6^,^36^

A separate study performed in peri-urban settlements in Cape Town, South Africa, investigated the prevalence of depressed mood during pregnancy in these populations.^6^ The study found that 39% of the pregnant women showed signs of depression. The psychosocial risk factors for maternal and postpartum depression include past history of mental illness, mental disturbance during pregnancy, family history of depression, low socio-economic status and poor interpersonal relationships.^38^ Postnatal depression is sometimes preluded by depression during pregnancy.^7^

## Depression as a potential risk factor for CVD during peripartum

Depression has been confirmed to be a risk factor for CVD in general.^30^ The mechanism by which depression is thought to contribute to the development of CVD is through an increase in oxidative stress, as well as inflammation.^33^ Oxidative stress and inflammation have both been suggested to contribute to the development of PPCM and pre-eclampsia.^26^,^29^ Depression during pregnancy may contribute to hypertension via excretion of vasoactive hormones.^39^ A prospective population study suggested that depression in early pregnancy was a risk factor for pre-eclampsia later in pregnancy.^39^ Depression has been linked to a higher risk of heart failure as well as poorer outcomes.^40^

**Fig. 2.**
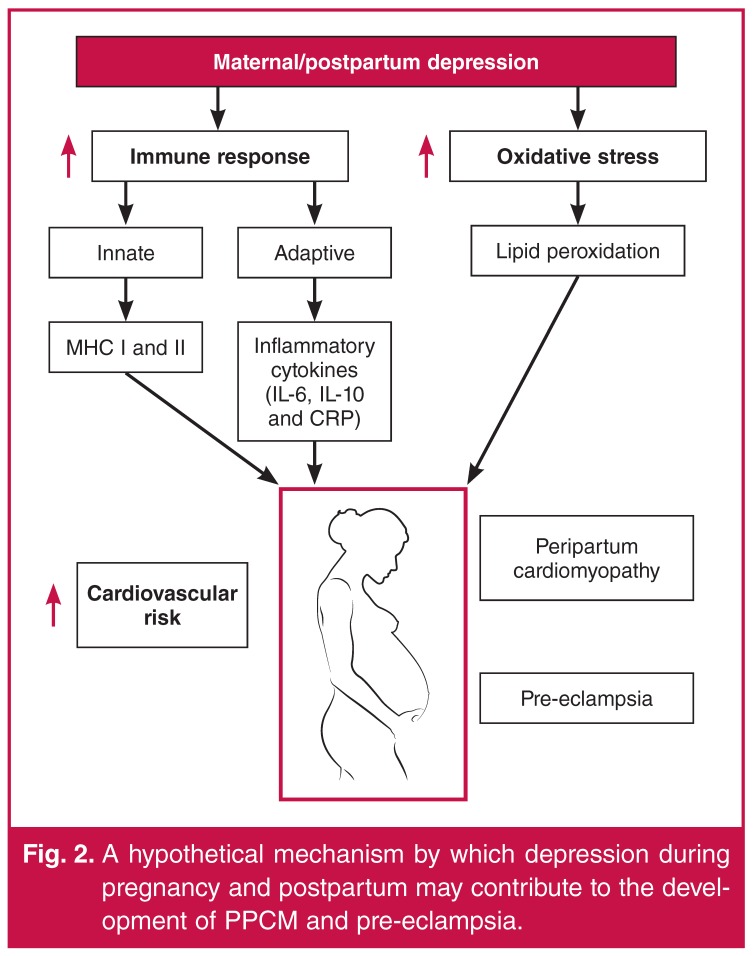
A hypothetical mechanism by which depression during pregnancy and postpartum may contribute to the development of PPCM and pre-eclampsia.

A hypothetical mechanism by which depression during pregnancy and postpartum may contribute to the development of PPCM and pre-eclampsia is shown in Fig. 2. The pathological increase in oxidative stress and inflammation caused by depression during the last trimester of pregnancy or postpartum may contribute to left ventricular heart failure in women with PPCM or hypertension in women with pre-eclampsia.

## The way forward

The aetiology of pregnancy-related cardiovascular complications in conditions such as pre-eclampsia and PPCM remain unclear. Depression during pregnancy and the postpartum period is a common condition. Previous studies have linked perinatal depression as a risk factor for pre-eclampsia. There is also evidence in the literature that depression is a risk factor for and a predictor of poor outcomes in CVD in general. The data have shown that depression causes an increase in the release of pro-inflammatory markers such as CRP and IL-6, which may contribute to the development of CVD, in particular PPCM. Further studies are required to determine whether depression in the peripartum period is indeed a risk factor for cardiovascular complications of pregnancy, for example, assessing the depression levels in a large group of pregnant women and then assessing their postnatal outcome.
